# Reverse Problem in Surface Texture Analysis—One-Process Profile Modeling on the Basis of Measured Two-Process Profile after Machining or Wear

**DOI:** 10.3390/ma12244169

**Published:** 2019-12-12

**Authors:** Pawel Pawlus, Rafal Reizer, Michal Wieczorowski

**Affiliations:** 1 Faculty of Mechanical Engineering and Aeronautics, Rzeszow University of Technology, Powstancow Warszawy 8 Street, 35-959 Rzeszow, Poland; ppawlus@prz.edu.pl; 2 College of Natural Sciences, University of Rzeszow, Pigonia Street 1, 35-310 Rzeszow, Poland; 3 Faculty of Mechanical Engineering and Management, Poznan University of Technology, Piotrowo Street 3, 61-138 Poznan, Poland; michal.wieczorowski@put.poznan.pl

**Keywords:** profile, two-process surface, correlation length

## Abstract

The method of the base (valley) one-process profile modeling on the basis of the measured two-process profile was developed. The base one-process random profile of the Gaussian ordinate distribution is characterized by the standard deviation of the profile height and the correlation length. The problem of estimation of the correlation length of this one-process profile exists. In the procedure of the correlation length estimation, information about the averaged shape of the autocorrelation functions of many one-process profiles after the same type of machining is required. The correlation length of the base one-process profile can be obtained on the basis of the vertical truncation of the measured two-process profile. The average error of the correlation length estimation was not higher than 7%, while the maximum error was not larger than 14%. This method can be extended to simulate the one-process texture of 3D (areal) surface topography.

## 1. Introduction

Many machined surfaces have an ordinate distribution similar to Gaussian distribution. They are called one-process surfaces because they contain tracks of only one machining process. Two-process surfaces can be created from initial one-process textures during a low wear (within the limits of the original surface topography). This type of the surface consists of smooth wear-resistant plateau parts with deep valleys working as reservoirs for lubricant and traps for wear debris. This structure connects in an ideal way the good sliding property of a smooth surface with the great ability to maintain oil of a porous structure. Two-process textures are more functionally important than one-process topographies.

Therefore, attempts were made to obtain a two-process surface during the last stage of machining. This surface should resemble a texture created during running-in. Due to it, a duration of running-in and wear decreased. For this reason, two-process textures were created. A plateau-honed cylinder surface is the practical example of such structures [1,2,3,4]. It consists of two random portions—smooth plateau and rough valley parts. Because of the excellent tribological properties of such surfaces, many other two-process textures were created—random, deterministic or random-deterministic structures. They are called textured surfaces. Surface texturing is an option of surface engineering resulting in significant reductions of the frictional resistance in mixed and fluid lubrications, wear, and the inclination to seizure by creating oil pockets (dimples or cavities) on the sliding surfaces [5,6,7,8,9,10].

**Figure 1:**
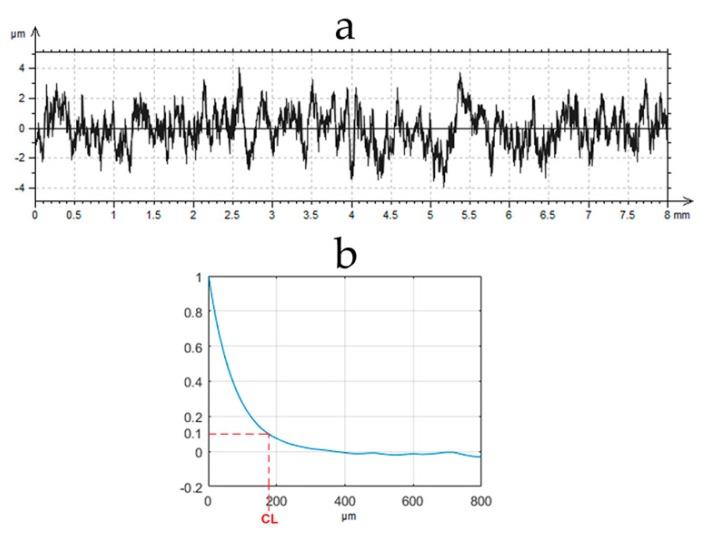
One-process random profile (**a**) and its autocorrelation function (**b**).

One-process surfaces are well described. The analysis of the measured one-process texture is simple. The digital filtration is an easy task. A typical Gaussian filter can be used. A one-process random profile can be easily described by only two parameters: height and horizontal. It can be completely characterized by the standard deviation of height (Pq), and a correlation length (CL), i.e., the distance at which the autocorrelation function slowly decays to a value of 0.1 [11,12]. Figure 1 presents the example of the random one-process profile and its autocorrelation function with the CL parameter.

A correct characterization of contact between rough surfaces is substantial during the study of tribological problems like friction, wear or sealing. However, the contact of surfaces of Gaussian ordinate distribution was typically analyzed [13,14,15].

Simulation of surface texture offers many advantages. Computer generations of surfaces created in manufacturing or wear processes lead to decreases in both cost and time of experimental research. The modeled surfaces can be used in various problems, such as contact, friction, and wear. A one-process profile can be modeled based on the values of the Pq parameter (standard deviation of roughness height) and the correlation length CL using the ARMA (autoregressive-moving average) [16] or FFT (Fast Fourier Transform) [17] methods.

However, there are some problems when analyzing two-process textures. The application of the Gaussian filter causes profile distortions near the edges of deep valleys. It is necessary to increase the cut-off or use different filters, like a double Gaussian filter (ISO 13565–1 [18]) or robust filter [19]. Description of a two-process profile is more complicated compared to that of a one-process profile. Two-process profiles cannot be described only by two parameters. Therefore, the special two standards dedicated for two-process surfaces occur. The first of them, ISO 13565–2 [20], is based on the profile division into three parts: peak, core, and valley. There are five parameters describing the material ratio curve of a two-process profile: the core roughness height Pk, the reduced peak height Ppk, the reduced valley depth Pvk, and two material ratios of transitions points between profile parts [21,22]. The second standard ISO 13565–3 [23] divides the profile into only two parts: peak (plateau) and valley; therefore, three parameters characterize the material ratio curve: Ppq (the plateau root–mean square roughness), Pvq (the valley root–mean square roughness), and Pmq (the material ratio of plateau–to–valley transition) [24,25]. However, both presented standards do not include horizontal parameters, which are functionally important [11,12,13,14,15].

## 2. Formulation of the Problem

**Figure 2:**
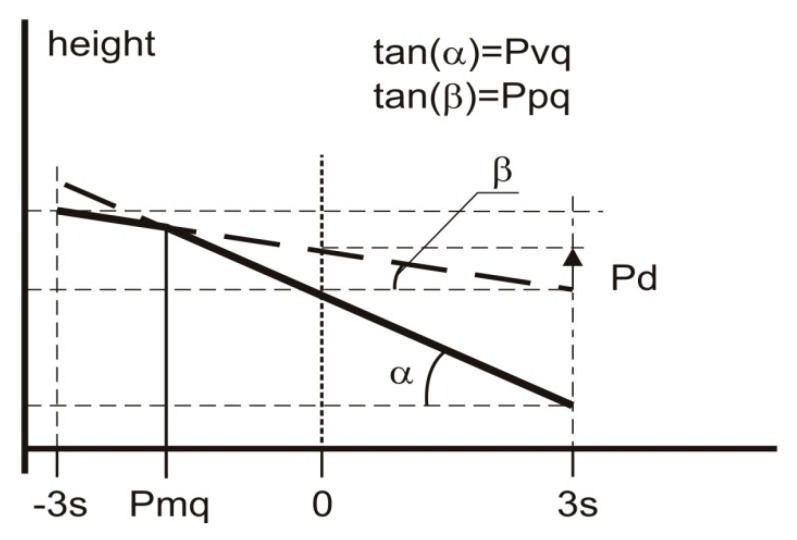
Graphical interpretation of the probability parameters.

The standard ISO 13565–3 [23] is the base of two-process profile modeling. The parameters describing the two-process surface can be calculated from a probability plot of the material ratio curve. The two-process profile is represented by two straight lines describing peak (plateau) and valley portions. The slopes of these lines are the standard deviation of plateau (Ppq) and of valley (Pvq) parts, respectively [24]. This standard also includes the Pmq parameter (Figure 2).

During simulation of the two-process profile, one should generate two Gaussian profiles (plateau and valley) and take the point-wise minimum [26,27]. More precisely, two profiles of Gaussian ordinate distributions are superimposed [26,27] for a given vertical distance between their mean lines Pd shown in Figure 2. The standard deviations of the plateau and valley profiles are equal to the Ppq and Pvq parameters of the two-process profile, respectively. The distance Pd relates to the parameters Ppq, Pvq, and Pmq by the following formula [26]:

(1) Pd=Pmq(Ppq−Pvq).

**Figure 3:**
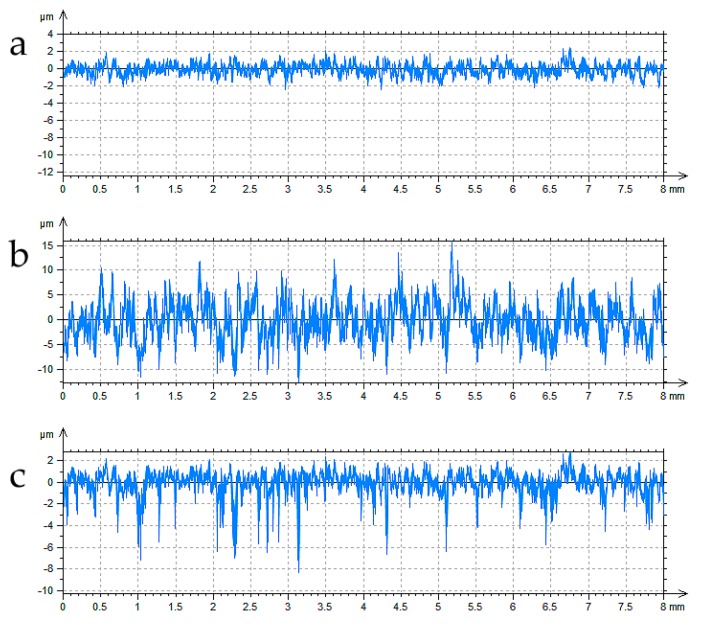
Computer creation of the two-process profile: plateau profile (**a**), valley profile (**b**), and two-process profile (**c**).

From ordinates of generated the two-process profile, the smaller ones are selected. Figure 3 presents the example of computer creation of the two-process profile.

Only amplitude parameters of plateau and valley profiles can be determined from the probability plot of the material ratio curve (Figure 3). However, each Gaussian profile is characterized not only by the height parameter (Sq), but also by the horizontal parameter, like the correlation length CL. They are necessary in the modeling procedure. The question arises of how horizontal parameters (correlation lengths) of two profiles of Gaussian ordinate distribution (plateau and valley) can be estimated. When these parameters are unknown, there would be a problem with obtaining the correct autocorrelation length of the two-process profile. The iterative procedure of selecting correlations lengths of plateau and valley Gaussian profiles can be a solution [28,29]. However, after its application, the correlation length of modeled two-process profile can be similar to that of the measured profile; nevertheless, the correlation lengths of plateau and valley parts can be incorrect.

Especially the proper estimation of the correlation length of the valley profile is the task of a primary importance, because this profile really exists, in contrast to the plateau profile. The profile after finish (one-step) honing during plateau honing and the one-process profile before wear are examples of valley profiles.

The following reverse problem should be solved: How can we simulate the valley profile (Figure 3b) when only the measured two-process profile is known (Figure 3c)? According to the knowledge of the authors of this paper, although many works in the field of surface topography modeling have been carried out, this problem has not been solved yet. Therefore, the solution to this problem is a novelty of this research work.

As mentioned above, each Gaussian profile can be described by the standard deviation of height (the Pq parameter) and the correlation length (CL). There is no problem with estimation of the Pq parameter of the valley profile. It is equal (close due to some measurement errors) to the Pvq parameter of the two-process profile. This parameter can be estimated from the probability plot of the material ratio curve (Figure 2).

The correct simulation of the valley profile is a problem of substantial importance. In some cases, one cannot measure the surface after machining, only after a low wear. It is necessary to obtain information about the shape of the one-process profile after machining to know how the roughness of the machined surface affects tribological properties of the sliding assembly. This problem is very important, since advanced machining methods have recently been used for surface topography creation [30,31,32,33,34]. Similar information is important also in a study of machining processes, like plateau honing. The question is how the initial surface roughness (after one-process finish honing) affects the surface obtained after the second process (plateau honing).

## 3. Solution of the Problem

The correlation length of the initial one-process profile can be estimated on the basis of analyses of (1) correlation lengths of many measured one-process profiles after the same type of machining treatment due to vertical truncation, and (2) the correlation length of the measured vertically truncated two-process profile, having only details belonging to the valley part.

**Figure 4:**
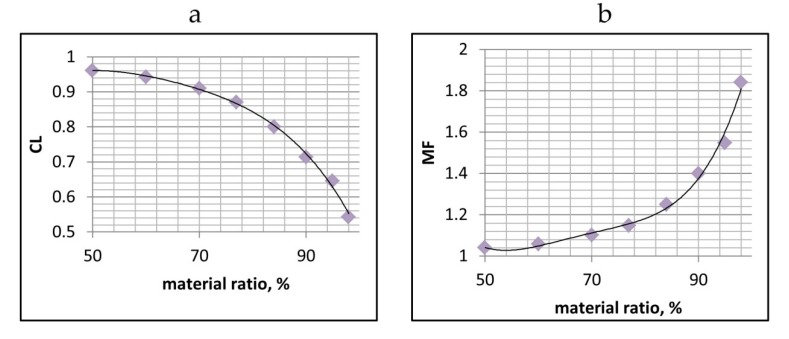
Correlation length (CL) of the truncated one-process profile after honing (**a**), and magnification factor (MF) (**b**) versus material ratio.

First, one should know how autocorrelation functions of one-process profiles after the same kind of machining are changing with vertical truncations of heights. It was found that due to truncation, the correlation length decreased. This was connected with reductions in the widths of deep valleys affecting the correlation length and the cumulative spectrum [35]. Because for one-process texture, the profile probability plot has the shape of the straight line, one should know how the profile correlation length changes for the given material ratio. After analyses of the set of profiles, the average value of the correlation length for selected material ratios should be computed. This analysis should be conducted for many surfaces after the same treatment, like polishing, lapping, grinding or abrasive blasting. For instance, Figure 4a presents the curve obtained for one-process surfaces after one-process finish honing of more than 20 cylinders, which were honed using various methods including different kinds of stones (diamond and ceramic). It is important that shapes of the ordinate distributions of these textures should be approximately symmetric—the skewness Psk should be near 0 and the emptiness coefficient Pp/Pt near 0.5, the Pq/Pa ratio near 1.25 [36] (Pp—maximum peak height, Pt—total profile height, Pa—arithmetic mean deviation of the profile), without spikes or individual valleys. Then, reciprocals of the obtained correlation lengths’ MF (magnification factors) for the given material ratios should be calculated (Figure 4b). The material ratios of truncated profiles were restricted to the 50–98% range.

Determination of the curve shown in Figure 4b on the basis of series of profiles after the same type of machining is the initial step.

**Figure 5:**
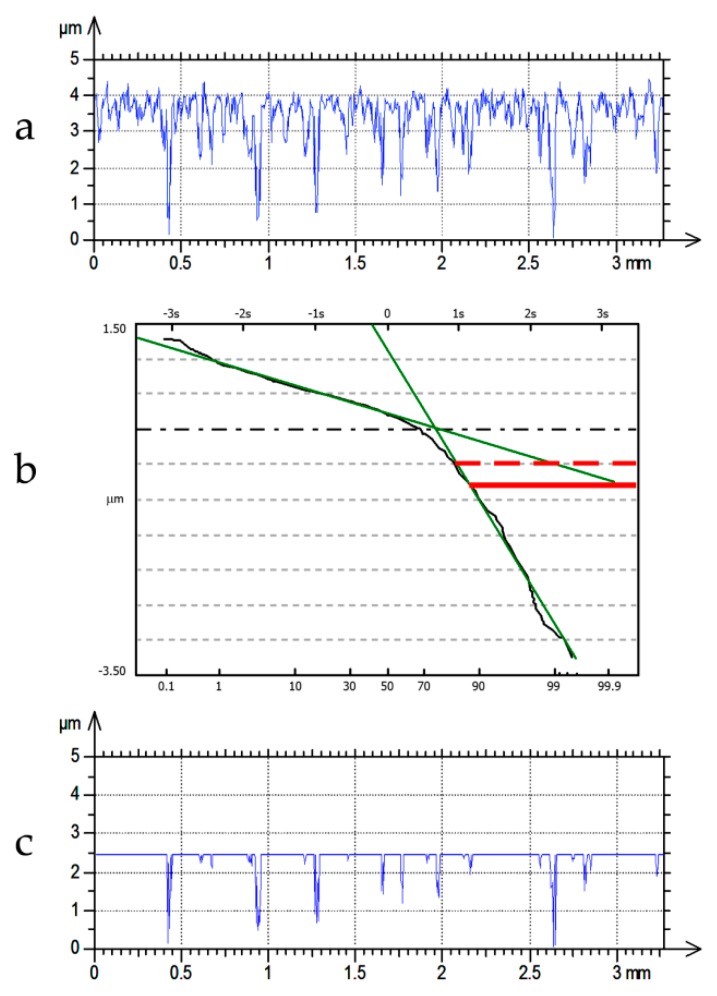
Two-process cylinder profile (**a**), its probability plot (**b**), and the truncated profile containing the valley part (**c**).

Then, for each measured two-process profile, it is necessary to extract details belonging only to the valley portion—Figure 5.

It is known that the part of the profile under the solid red line originates from the valley profile (Figure 5b). Therefore, the two-process profile (Figure 5a) should be vertically truncated and its lower part ought to be analyzed. In this case, the truncation level is the ordinate of crossing the approximate straight line characterizing the valley parts with the right vertical axis (in a profile study, it is a little larger than 3 s or 99.87%). However, sometimes, for a profile characterized by a high transition material ratio Pmq (Figure 2), the valley part could contain mainly individual nonstatistical valleys. In this case, the truncation level should lie at the top (see dotted line in Figure 5b).

However, one should know that near the transition point (of the abscissa Pmq), there can be a mixture of profile details originating from both the plateau and valley portions, which could be a source of the error. Therefore, the selection of the truncation level should be treated with great care.

The correlation length of the one-process valley profile is equal to the correlation length of the truncated two-process profile (Figure 5c) multiplied by the magnification factor (MF) obtained from Figure 4b for the given material ratio of the truncation level (Figure 5b).

After determination of the curve presenting the magnification factor MF versus the material ratio (Figure 4b) on the basis of series of profiles after the same type of machining, there are the following steps in the procedure of simulation of the profile of the base (valley) one-process texture:

- Determination of the probability plot of the two-process measured profile;
- Determination of the Pq parameter of the one-process profile which is the Pvq parameter of the two-process profile;
- Vertical truncation of the two-process profile to extract profile details belonging only to the valley portion;
- Determinations of the autocorrelation function and correlation length of the truncated two-process profile;
- Calculation of the magnification factor MF (Figure 4b) based on the material ratio of the truncation level;
- Calculation of the correlation length of the one-process valley profile by magnification of the correlation length of the truncated two-process profile by the MF value;
- Simulation of the base (valley) profile using for example the FFT method [17].

**Figure 6:**
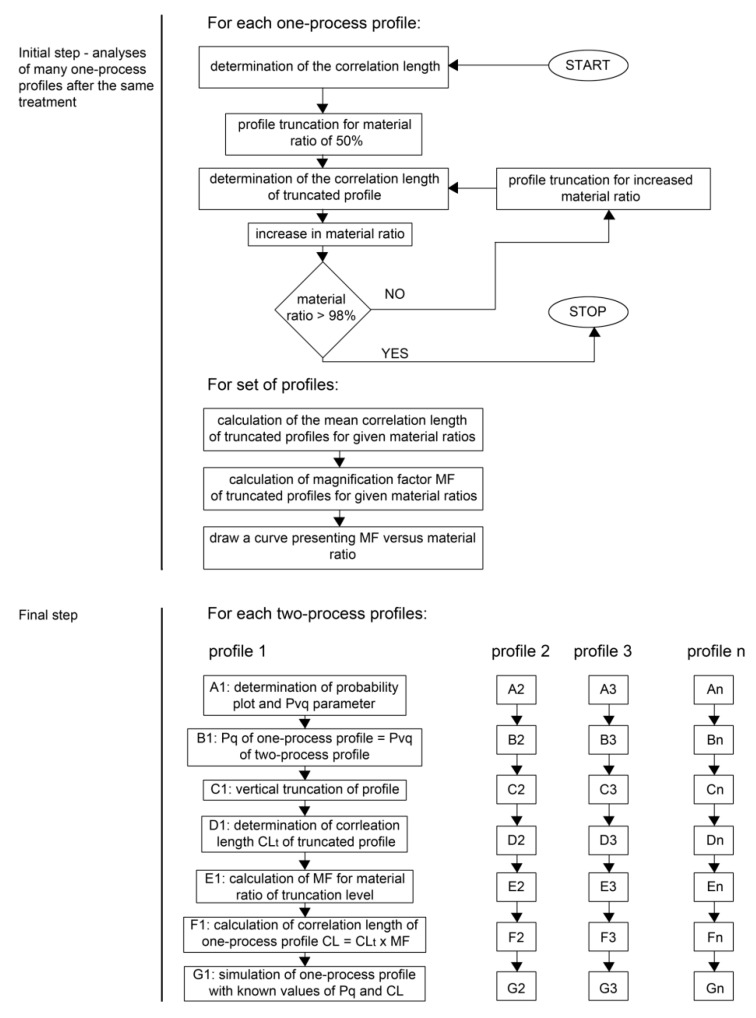
Methodology of one-process base (valley) profile simulation.

Figure 6 presents a flow chart of research methodology. One can see that on the basis of a graph presenting the magnification factor MF versus the material ratio (Figure 4b), many profiles can be simulated.

## 4. Validation of Method

**Figure 7:**
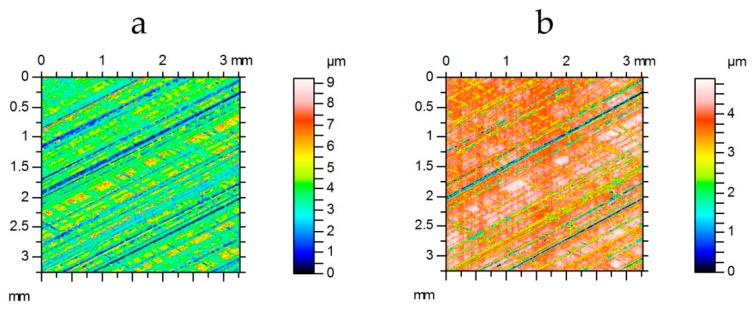
Surface of cylinder liner after finish honing (**a**) and after the tribological test (**b**).

This method was validated for two groups of cylinder liner surfaces made from grey cast iron. Cylinder liners from the first group were only finish honed with diamond or ceramic tools. Therefore, they had one-process random textures. These cylinders were subjected to tribological tests using an OPTIMOL SRV5 tester (Optimol Intruments, Munich, Germany). They co-acted with details of piston rings under lubricated conditions. During these tests, a low wear took place. Before and after test topographies of cylinder liner surfaces were measured in very similar places (the mechanical and then digital relocations were used) by a white light interferometer Talysurf CCI Lite—Figure 7. The initial measuring area of 3.3 × 3.3 mm contained 1024 × 1024 points. During the analysis, the form was removed by the polynomial of the second level; a digital filtration was not used. Spikes were eliminated by truncation.

From the surfaces of the cylinders, axial profiles were selected. For each profile after operating, the procedure described above was conducted for at least three truncation levels, starting from the upper level resulting from the probability plot of the material ratio curve (Figure 5b). The lowest level typically corresponded to material ratio of 98%; however, this level depended on the shape of the material ratio curve. For the one-process cylinder liner profile after finish honing, the correlation length was computed. The average correlation length of the profile after finish honing estimated from truncated cylinder liner profile after operating was compared with that from the measured (real) profile. About 20 different surface textures were analyzed. It was found that during operating, only the wear removal took place, without occurrence of the plastic deformation.

**Figure 8:**
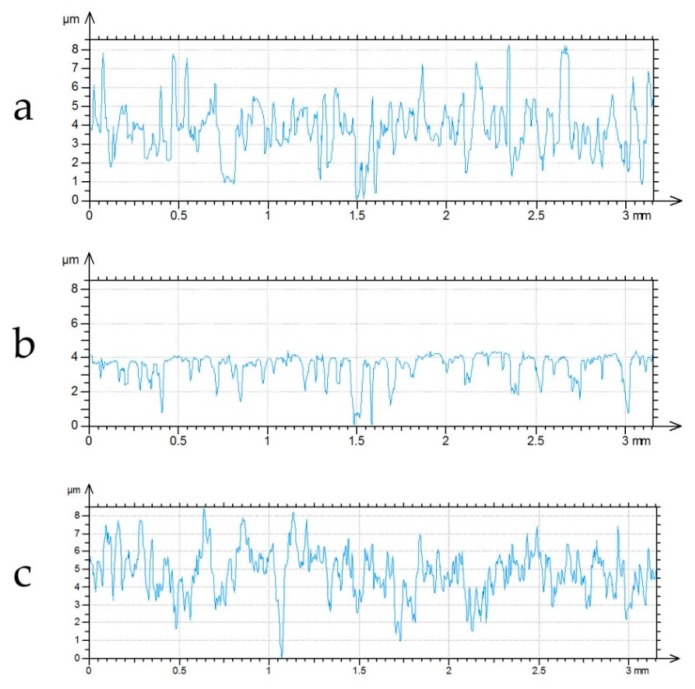
One-process cylinder liner profile after finish honing (**a**), after tribological test (**b**), and simulated one-process profile (**c**).

It was found that the average error of estimation of the correlation length of profiles after finish honing was 6.5%, while the maximum error was 12%. Figure 8 presents the example of one-process, two-process, and modeled one-process profiles. The one-process profile was simulated using the FFT method [17].

Similar tests were carried out for cylinders from the second group. There were finish honed (one-process) and plateau honed (two-process) surfaces of cylinder liners. In contrast to cylinders from the first group, the measurements of various one-process and two-process cylinder liners were conducted in similar places. However, the honing treatment of one-process and finish honing of two-process cylinder liners were carried out under the same conditions (see [37]). For each surface (also measured by the white light interferometer), three axial profiles were selected. The test procedure was similar to that mentioned above; however, the average value of three correlation lengths from one-process cylinder profiles (after finish honing) was compared with the average value of estimated correlation length obtained on the basis of measurement of three two-process profiles after plateau honing. It this part of study, about 20 different surface topographies were analyzed.

**Figure 9:**
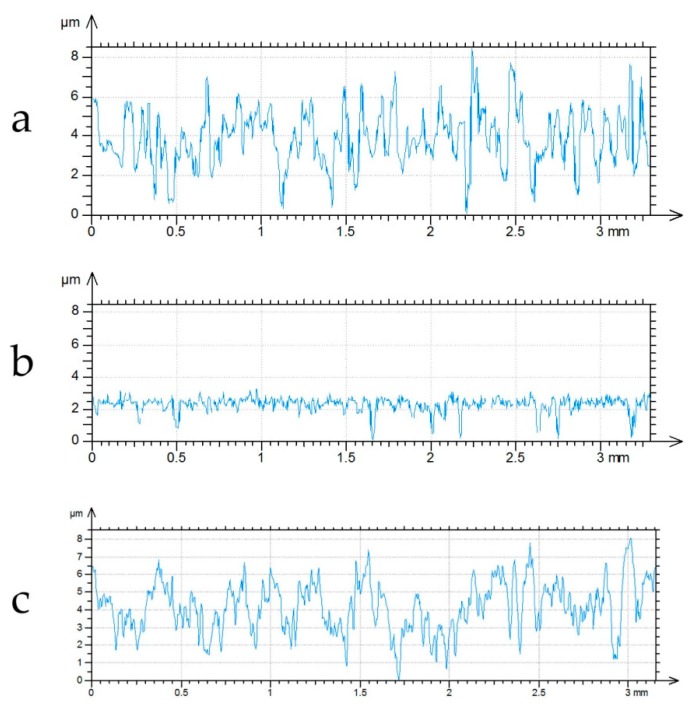
One-process cylinder liner profile after finish honing (**a**), after plateau honing (**b**), and the simulated one-process profile (**c**).

It was found that the mean error of estimation of correlation lengths of profiles after finish honing was 5.8%; however, the maximum error was 15%. Figure 9 shows the example of one-process, two-process, and simulated one-process profiles.

## 5. Discussion

One can see that the application of this method assured good results in both analyzed cases. The authors of this paper focused mainly on estimation of the correlation length, because the standard deviation of the roughness height can be obtained from the probability plot of the material ratio curve of two-process surfaces (Figure 5b). However, there can be errors connected with its estimation. For instance, the valley part in the roughness probability plot of a two-process surface sometimes has a deviation from the linear shape. However, these errors were analyzed elsewhere.

There are some limitations to the presented method application. The first of them is mentioned above: the necessity to exclude profile details related to individual nonstatistical grooves and the mixture of two processes. Therefore, the determination of the truncation higher level (Figure 5b) should be treated with great care. When it is too high (corresponding to a low material ratio), the valley part can include the mixture of the two processes. However, when it is too low (corresponding to a high material ratio), the individual grooves can disturb the results of one-process profile modeling. On the basis of analyses of many surfaces, the lowest truncation level for the material ratio of 98% was selected (Figure 4 and Figure 6). The precise analysis of the probability plot of the material ratio curve can be helpful. The remaining valley part should not be changed during wear or machining. Therefore, the conditions of wear and machining processes should be carefully taken into consideration. Especially during wear, sometimes plastic deformation, not only wear removal, can take place. The correct determination of the correlation length can be the other problem. Although the effect of the sampling interval on the correlation length is low, the sampling interval should be small enough for the correct estimation of the correlation length (smaller than 0.4 of the correlation length) [38].

This method can be also used in 3D (areal) analysis of isotropic or anisotropic one-directional surfaces. In this case, the correlation lengths in perpendicular directions should be determined on the basis of analyses of several representative profiles.

The correlation length of the upper plateau profile can also be estimated using this method. However, this task is not such important as the analysis of the valley profile, since the plateau profile does not really exist. However, information of the correlation lengths of both plateau and valley parts can be helpful in the simulation of two-process profiles or 3D textures.

This method was dedicated to the random base profile. When the valley part of two-process surface has a deterministic character (piston skirt after a low wear [39] can be the example), it can be modeled more easily compared to the random valley portion [12].

## 6. Conclusions

The method of simulation of the one-process base (valley) profile on the basis of the two-process profile was developed. The one-process profile is characterized by the standard deviation of the height and the correlation length. In the simulation procedure, the probability plot of the two-process profile is helpful. After analysis of this plot, the height of the one-process profile can be directly estimated. The correlation length can be achieved based on the vertical truncation of the two-process profile. In the procedure of correlation length, estimation information about autocorrelation function shapes of many profiles after the same kind of machining is needed.

The proposed procedure was validated for two groups of surfaces. It was found that the average error of the correlation length of one-process profile estimation was not higher than 7%, while the maximum error was not larger than 14%.

This method can be easily extended to simulate the base one-process isotropic or one-directional anisotropic 3D (areal) surface topography. It would be helpful in two-process surface modeling.

## References

[1] Grabon W., Pawlus P., Wos S., Koszela W., Wieczorowski M. (2017). Effects of honed cylinder liner surface texture on tribological properties of piston ring-liner assembly in short time tests. Tribol. Int..

[2] Haasis G., Weigmann U. (1999). New honing technique reduces oil consumption. Ind. Diam. Rev..

[3] Mezghani S., Demirci I., Zahouani H., El Mansori M. (2012). The effect of groove texture patterns on piston-ring pack friction. Precis. Eng..

[4] Anderberg C., Dimkovski Z., Rosén B.G., Thomas T.R. (2019). Low friction and emission cylinder liner surfaces and the influence of surface topography and scale. Tribol. Int..

[5] Gachot C., Rosenkranz A., Hsu S.M., Costa H.L. (2017). A critical assessment of surface texturing for friction and wear improvement. Wear.

[6] Rosenkranz A., Grützmacher P.G., Gachot C., Costa H.L. (2019). Surface Texturing in Machine Elements: A Critical Discussion for Rolling and Sliding Contacts. Adv. Eng. Mater..

[7] Koszela W., Dzierwa A., Galda L., Pawlus P. (2012). Experimental investigation of oil pockets effect on abrasive wear resistance. Tribol. Int..

[8] Galda L., Dzierwa A., Sep J., Pawlus P. (2010). The effect of oil pockets shape and distribution on seizure resistance in lubricated sliding. Tribol. Lett..

[9] Koszela W., Pawlus P., Reizer R., Liskiewicz T. (2018). The combined effect of surface texturing and DLC coating on the functional properties of internal combustion engines. Tribol. Int..

[10] Mishra P., Ramkumar P. (2019). Effect of additives on a surface textured piston ring–cylinder liner system. Tribol.-Mater. Surf. Interfaces.

[11] Whitehouse D.J., Archard J.F. (1970). The properties of random surface of significance in their contact. Proc. R. Soc. (Lond.).

[12] Whitehouse D.J. (1994). Handbook of Surface Metrology.

[13] Greenwood J.A., Williamson J.B.P. (1966). Contact of nominally flat surfaces. Proc. R. Soc. (Lond.).

[14] Pawlus P., Zelasko W. (2012). The importance of sampling interval for rough contact mechanics. Wear.

[15] An B., Wang X., Xu Y., Jackson R.L. (2019). Deterministic elastic-plastic modelling of rough surface contact including spectral interpolation and comparison to theoretical models. Tribol. Int..

[16] Teja S.R., Jayasingh T. (1993). Characterisation of ground surface profiles: A comparison of AR, MA and ARMA modelling methods. Int. J. Mach. Tools Manuf..

[17] Wu J.J. (2000). Simulation of rough surfaces with FFT. Tribol. Int..

[18] (1996). ISO 13565-1—Geometrical Product Specifications (GPS)—Surface Texture: Profile METHOD; SURFACES HAVING Stratified Functional Properties—Part 1: Filtering and General Measurement Conditions.

[19] Li H., Jiang X., Li Z. (2004). Robust estimation in Gaussian filtering for engineering surface characterization. Precis. Eng..

[20] (1996). ISO 13565-2—Geometrical Product Specifications (GPS)—Surface Texture: Profile method; Surfaces having Stratified Functional Properties—Part 2: Height Characterization Using the Linear Material Ratio Curve.

[21] Bohm H.J. (1992). Parameters for evaluating the wearing behaviour of surfaces. Int. J. Mach. Tools Manuf..

[22] Schneider U., Steckroth A., Rau N., Hubner G. (1988). An approach to the evaluation of surface profiles by separating them into functionally different parts. Surf. Topogr..

[23] (1998). ISO 13565-3—Geometrical Product Specifications (GPS)—Surface Texture: Profile Method; Surfaces Having Stratified Functional Properties—Part 3: Height Characterization Using the Material Probability Curve.

[24] Malburg M.C., Raja J. (1993). Characterization of surface texture generated by plateau-honing process. CIRP Ann..

[25] Whitehouse D.J. (1985). Assessment of surface finish profiles produced by multiprocess manufacture. Proc. Inst. Mech. Eng..

[26] Pawlus P. (2008). Simulation of stratified surface topographies. Wear.

[27] Pérez-Rafols F., Almqvist A. (2019). Generating randomly rough surfaces with given height probability distribution and power spectrum. Tribol. Int..

[28] Reizer R., Pawlus P. (2012). Modeling of plateau honed surface topography. Proc. Inst. Mech. Eng. Part B J. Eng. Manuf..

[29] Hu S., Brunetiere N., Huang W., Liu X., Wang Y. (2017). Bi-Gaussian surface identification and reconstruction with revised autocorrelation functions. Tribol. Int..

[30] Kishawy H.A., Hegab H., Umer U., Mohany A. (2018). Application of acoustic emissions in machining processes: Analysis and critical review. Int. J. Adv. Manuf. Technol..

[31] Zhu L., Yang Z., Li Z. (2019). Investigation of mechanics and machinability of titanium alloy thin-walled parts by CBN grinding head. Int. J. Adv. Manuf. Technol..

[32] Zhao B., Ding W., Chen Z., Ya C. (2019). Processes Pore structure design and grinding performance of porous metal-bonded CBN abrasive wheels fabricated by vacuum sintering. J. Manuf. Process..

[33] Meng B., Yuan D., Xu S. (2019). Coupling effect on the removal mechanism and surface/subsurface characteristics of SiC during grinding process at the nanoscale. Ceram. Int..

[34] Krolczyk G.M., Maruda R.W., Krolczyk J.B., Wojciechowski S., Mia M., Nieslony P., Budzik G. (2019). Ecological trends in machining as a key factor in sustainable production: A review. J. Clean Prod..

[35] Pawlus P., Chetwynd D.G. (1996). Efficient characterization of surface topography in cylinder bores. Precis. Eng..

[36] King T.G., Spedding T.A. (1982). On the relationship between surface profile height parameters. Wear.

[37] Pawlus P., Dzierwa A., Michalski J., Reizer R., Wieczorowski M., Majchrowski R. (2014). The effect of selected parameters of the honing process on cylinder liner surface topography. Surf. Topogr.-Metrol. Prop..

[38] Pawlus P. (2007). Digitisation of surface topography measurement results. Measurement.

[39] Krzyzak Z., Pawlus P. (2006). ‘Zero-wear’of piston skirt surface topography. Wear.

